# Operating theatre nurses' with managerial responsibility: Self‐reported clinical competence and need of competence development in perioperative nursing

**DOI:** 10.1002/nop2.1120

**Published:** 2021-11-06

**Authors:** Ann‐Catrin Blomberg, Lillemor Lindwall, Birgitta Bisholt

**Affiliations:** ^1^ Department of Health Sciences Karlstad University Karlstad Sweden; ^2^ Department of Health Sciences Swedish Red Cross University College Huddinge Sweden

**Keywords:** competence, competence development, operating theatre nurse, perioperative nursing

## Abstract

**Aim:**

The aim of this study was to investigate operating theatre nurses (OTNs) with managerial responsibility, and their self‐rated clinical competence and need for competence development in perioperative nursing.

**Design:**

A cross‐sectional study was applied using a modified version of Professional Nurse Self‐Assessment Scale of Clinical Core Competence I.

**Method:**

Data were collected from 303 OTNs in Sweden, 80 of whom indicated that they had managerial responsibility. Statistics analysis was used to identify the relationships between background variables to compare OTNs with and without managerial responsibility and their need for competence development.

**Results:**

OTNs with an academic degree and managerial responsibility self‐rated their clinical competence higher compared with OTNs without an academic degree. It also turned out that OTNs with RN education and 1‐year advanced nursing in theatre care, and master's 60 credits had a lower need for competence development in cooperation and consultation, professional development and critical thinking.

## 1 INTRODUCTION

In recent years, conditions in perioperative nursing in Sweden have changed towards increased demand for efficiency and a push for continuity in patient care in an increasingly high‐tech environment (Blomberg et al., 2014). This puts demands on operating theatre nurses (OTNs) with managerial responsibility. Huston (2008) argued that nurses with management responsibility positions do not have the qualifications required in a complex environment. Leading complex healthcare environments require a more global perspective and thinking. This is to develop nursing, the ability to integrate technology that facilitates mobility and portability of relationships, interactions and operational processes as well as decision‐making based on empirical science. Another aspect worth noting is that in perioperative care in Scandinavia, registered nurses (RNs) with long professional experience are recruited to have managerial responsibility and they are not prepared for what the assignment entails (Solbakken et al., 2020). This was also shown internationally, as was the fact that there was a lack of education in leadership, although OTNs were expected to meet the requirements and responsibilities of being a leader (Beitz, 2019; McCallin & Frankson, 2010; Pilat & Merriam, 2019). During the last year, perioperative care development has increased in Sweden (Blomberg et al., 2018a, 2018b), patient flow has increased and OTNs claims that they are not given the opportunity to provide the patient with the care they need. This requires additional education in leadership. Even in nursing education, leadership training is deficient (Francis‐Shama, 2016). In Sweden, today there are OTNs with managerial responsibility with varying education and professional experience in perioperative nursing, with or without an academic degree and formal academic leadership education (Blomberg et al., 2019). Despite this, demands are made that they should be able to take responsibility for developing and leading the perioperative practice, while having limited knowledge of leadership, usually given during ongoing assignments. This study was how OTNs perceive their clinical competence in leadership and need for competence development to be able to take full managerial responsibility.

## 2 BACKGROUND

Within perioperative nursing, OTNs are required to have specific clinical competence to be able to work independently and to cooperate in a surgical team to ensure patient safety. In this study, perioperative nursing is described in relation to Swedish conditions, presented by Blomberg et al. (2018): Perioperative nursing is a nurse anaesthetists’ and operating theatre nurses’ pre‐, intra‐ and postoperative care for a patient who is undergoing surgery’. Perioperative nursing includes all nursing activities related to the surgical treatments, organization and leadership of the perioperative practice. OTNs with managerial responsibility require skills, knowledge and qualifications in care. The managerial responsibility is described according Wei et al. (2019) and Jangland et al. (2017) to be responsible for both the employers work environment and patients’ care to ensure the right competence to achieve a high quality of care. In addition, patients believe that nurses should be competent and that they have interpersonal skills (Smith, 2012).

The International Council of Nurses (ICN) emphasizes that the manager has an ethical responsibility to respect human rights, consider human values, to develop and watch over the safety of patients and staff environment (International Council for Nurses, 2013). The definition of competence is inconsistent. It has changed over time and varies in different countries (Gunawan et al., 2020). The meaning of the concept of competence can be understood in different ways based on the eye of the beholder. In this study, the meaning of competence is based on Ellström’s (1992) definition of having the ability to act and perform the specifics of duty in a certain situation and to reflect on and critically analyse and evaluate one's own way of carrying out the work. Clinical competence is not only made up of education and professional experiences, but also the ability to integrate theory and practice. The provision of health professionals with the right competence is the care provider's responsibility and includes both technical and non‐technical skills, as well as opportunities for competence development to be able to take responsibility and ensure patient care (SFS, 2017:30). Some studies have described OTNs core competencies in Sweden (Falk‐Brynhildsen et al., 2019; Jaensson et al., 2018), it shows that OTNS rated their competence lower in the factor empathy in caring relationship with the patient. OTNS rated itself higher in leadership compared to anaesthesia nurses.

Competence development is described from the technical‐rational perspective where the focus is on achieving the organization's goals, while a humanistic perspective is based on a personal need to achieve the requirements set for the assignment, for example, the development of leadership (Ellström, 1992). Competence development in perioperative nursing focuses more on medical technology and needs to be supplemented with education in perioperative nursing care (Blomberg et al., 2019). Smith and Palesy (2018) also believe that there is a risk that competence in medical technology is given priority over nursing care. Bull and Fitzgerald (2006) pointed out the importance of combining medical technology with caring to ensure patient safety.

Operating theatre nurses with managerial responsibility must be prepared to relate to organizational changes and create a sense of community in the workplace, where people can experience unity and fellowship of caring while fighting to improve quality of care despite financial pressures (Rudolfsson & Flensner, 2012; Rudolfsson et al., 2007). Within perioperative practice in Sweden, there are OTNs with different managerial responsibility as a first‐line manager, nurse assistant manager, section leader and function responsibility. The first‐line manager work closely with patients and employees and have personal responsibility and the assisting nurse manager assists with administrative tasks and practical support for the nurse manager so that they can focus on larger issues and responsibilities. Then, there are also OTNs with responsibility in various sections (e.g. orthopaedics and gynaecology) and function (medical technology and hygiene), within perioperative practice, and all of them have different leadership styles.

Donnelly (2017) described different leadership styles in perioperative practice and states that a leadership role is assumed by those wishing to initiate change. The transactional leader is the autocratic leader and considered to be controlling with a focus on goal achievement, unlike the transformational leader who has less overall control and encourages employees to be involved in decision‐making. It is essential that nurse managers can develop different leadership styles, appropriate to the demands of the situation (Donnelly, 2017). Bondas (2003) illustrates that nurse managers care about patient needs and describes five relationship‐based rooms that each represent one aspect of leadership.

Caritative leadership is, according to Solbakken et al. (2018), about nurturing and growing relationships to safeguard the best nursing care. However, it is a challenge to be a nurse manager, and Sørensen et al. (2011) explain that there is sometimes a conflict between the desire to be clinical and at the same time not being clinical enough, because the managerial responsibility must be prioritized. This leads to being tied up with administrative, managerial and financial duties. Therefore, it is important to investigate how OTNs self‐rated their managerial responsibility, and if they need further competence development. The aim of this study was to investigate OTNs with managerial responsibility, and their self‐rated clinical competence and need for competence development in perioperative nursing.

## 3 THE STUDY

### 3.1 Design and settings

This study is part of a larger research project aiming to investigate OTNs’ competence and need for competence development. A cross‐sectional study design was used and conducted in 2016 in Sweden. A questionnaire was used for data collection and all clinically active OTNs working in operating theatres in university, regional/central and district hospitals were invited to participate. Sweden is divided into six medical regions, which organize highly specialized care based on need and availability. In each region, there is a university hospital that besides advanced health care also conducts research and education. Regional/county hospitals offer various general surgical specialties and district hospitals have fewer surgical specialties. In perioperative care, there are active OTNs with different educational backgrounds as OTNs, from 2‐year direct education without postgraduate education in theatre care to RNs with 1‐year advanced nursing in theatre care and master's degree of 60 credits (Table 1).

### 3.2 Participants

A convenience sampling was used. In total, 1,057 OTNs were asked to participate, and 303 answered. One of the background variables in the questionnaire was experience of managerial responsibility, which 80 indicated that they had managerial responsibility (Blomberg et al., 2019). They were employed at university, regional/central and district hospitals and had varying lengths of work experience in perioperative care and managerial responsibility. They also had different educational background to become OTNs.

### 3.3 Instrument and measurement

The modified Professional Nurse Self‐Assessment Scale of clinical competence in perioperative nursing (PROFFSNurse SAS) was used for data collection in the present study. The PROFFSNurse SAS was chosen because it focuses on dynamic and mutual nurse–patient relationships and the theoretical foundation is based on Aristoteles three dimensions of knowledge (episteme, techne and phronesis). It is based on PROFFSNurse SAS I, which in turn, is based on the Nurse Clinical Competence Scale (NCCS). It was tested psychometrically in home care contexts in Norway (Finnbakk et al., 2015). A pilot test was performed on the questionnaire to see if it would be used internationally in the self‐assessment of nurses in postgraduate education and named Professional Nurse SAS II (Taylor et al., 2020; Wangensteen et al., 2018). Prior to use in perioperative nursing, its applicability was ensured by the research team (ACB, LL and BB).

A pilot test was conducted with 10 OTNs from various operating theatres in Sweden. As a result, 10 items were excluded as included clinical assessment of the patient's diagnosis and counselling on prevention and rehabilitation, which were not relevant in perioperative nursing. Approval of the revision was performed by the research group who developed the PROFFSNurse SAS I (Finnbakk et al., 2015). The modified version contained 43 items across six components: Direct clinical practice (15 items), covers important aspects of OTN clinical practice at different educational levels. Professional development (five items) includes being able to take personal responsibility for competence and professional development, as well as participation in quality and improvement work. Ethical decision‐ making (10 items) includes ethical values that take care of patients’ physical, social, mental and spiritual needs, as well as moral commitment in relation to OTNs’ clinical competence. Clinical leadership (four items) includes communicating and taking personal responsibility to make their own decisions as well as being aware of the consequences. Cooperation and consultation (six items) are about cooperation in the surgical team and the need for consultation with other professionals to ensure patient care. Finally, critical thinking (three items) includes how OTNs integrate scientific knowledge into perioperative practice to provide patient safety. The Cronbach's alpha varied between 0.78–0.97 (Table 2). Each item was asked referring to self‐assessment of clinical competence and the need for further competence development. The rating scales range from 1–10, where 1 indicates a low level and 10 indicates high levels.

### 3.4 Data collection

Data were collected digitally from the modified questionnaire PROFFSNurse version in perioperative nursing. An inquiry about study participation was made to all heads of departments in all 21 regions/counties in Sweden. One county did not reply and was, therefore, excluded. After the heads of departments had given approval of the study, a contact person was appointed to gain access to the participants’ email addresses. When the participants answered, they also gave their informed consent to participate. A total of four reminders were included and these were sent at 14‐day intervals.

### 3.5 Data analysis

The analysis of quantitative variables was carried out by calculating participants’ background such as age, educational background, academic degree, place of employment and professional experience in perioperative nursing, and experience of managerial responsibility (Table 1). Analytical statistics were performed by analysis of variance (ANOVA) to search for relationships between background factors and the six components of OTNs with or without managerial responsibility. Background factors were tested between main and backgrounds between two factors, both in terms OTNs’ self‐assessment of clinical competence as well as competence development needs. When statistically significant items were detected, a post hoc test analysis was performed using Fisher's LSD to investigate which group differed from the others in each component. The level of statistical significance was set to α = 0.05. Data were analysed using IBM SPSS the Statistic 25.

## 4 RESULTS

### 4.1 Characteristics of the study group

The study included 80 OTNs who had various managerial responsibilities. The average age was about 50 years and 49% of the participants stated that they had <5 years of experience of managerial responsibility. Half of the participants had >20 years of professional experience in perioperative nursing. The participants had different educational backgrounds to OTN, where 30% had a direct education without postgraduate education in theatre care, and more than half no academic degree. The distribution among universities, regional/central and district hospitals was about the same (Table 1). The means and standard deviation, as well as how OTNs rated their managerial responsibility regarding clinical competence in the six components is shown in Table 2. The result includes two parts. The first is a comparison of self‐assessment of clinical competence between OTNs with or without managerial responsibility. Then follows a description of the needs for competence development in their managerial responsibility.

**TABLE 1:** Demographic background of OTNs with managerial responsibility (n = 90)

Demographic characteristics	*n* (%)
Gender	
Female	82 (92.1)
Male (Missing 1)	7 (7.9)
Age (years)	
Mean score (*SD*)	49.85 (7.78)
Range (Missing 4)	31–62
Educational background	
2‐year direct education and no postgraduate in theatre care	42 (47.7)
RN education with 1‐year postgraduate in theatre care	28 (31.8)
RN education with 1‐year advanced nursing in theatre care (Missing 2)	18 (20.5)
Academic degree	
Bachelor degree	20 (23)
Master degree (60 credits)^a^	14 (16.1)
Ingen (Missing 3)	53 (60.9)
Professional experiences in perioperative nursing	
<10 years	13 (14.6)
10–20 years	30 (33.7)
>20 years (Missing 1)	46 (51.7)
Place of employment	
University hospital	30 (34.1)
Regional hospital	29 (33)
District hospital (Missing 1)	29 (33)
Managerial responsibility	
First‐line manager	16 (20)
Assisting nurse manager	7 (8.8)
Responsibility section leader	38 (47.5)
Function responsibility (Missing 10)	19 (23.8)
Experience of managerial responsibility	
<5 years of experience	44 (48.9)
>5 years of experience	46 (51.1)

Abbreviation: RN, Registered Nurse.

^a^ Master degree (60 credits) correspond to 1 year at Master program.

**TABLE 2:** Descriptive statistic for the six components of the modified PROFFSNurse SAS in perioperative nursing (*n* = 80)

	PROFFS Nurse SAS components	Number of items	Mean	*SD*	Observed range Cronbach's α
Direct clinical practice	15	114.4	16.3	67–148	0.97
Professional development	5	40	7	21–50	0.96
Ethical decision‐making	10	75.6	11.8	51–99	0.97
Clinical leadership	4	36.3	3	25–40	0.95
Cooperation and consultation	6	52.2	6	35–60	0.95
Critical thinking	3	23.8	4.3	8–30	0.78

#### 4.1.1 Comparing between OTNs with and without managerial responsibility regarding clinical competence

Operating theatre nurses with managerial responsibility was statistically significant in *direct clinical practice, professional development and critical thinking* (Table 3). OTNs with managerial responsibility (α = 0.001) rated themselves higher in *professional development* compared to those without managerial responsibility. Analysis of OTNs with managerial responsibility revealed a significant interaction between having an academic degree and having managerial responsibility, regarding *direct clinical practice and critical thinking*. Participants with master's degree of 60 credits rated themselves much higher in direct clinical practice (α = 0.002) compared to those with a bachelor's degree. Participants with master's 60 credits also self‐rated higher (α = 0.015) regarding *critical thinking*, but this was only a marginal increase compared with those with a bachelor's degree. OTNs with managerial responsibility and no academic degree rated themselves low in both *direct clinical practice and critical thinking*. When it came to *professional developmen*t, a statistical significance emerged between managerial responsibility and education to become an OTN, for participants with managerial responsibility. OTNs with RN education and 1‐year advanced nursing in theatre care rated themselves higher compared to others.

**TABLE 3:** Comparing clinical competence regarding OTN’s with (*n* = 80) and without a managerial responsibility (*n* = 201)

The six components of the modified PROFFNurse SAS in perioperative nursing	Clinical leadership			Cooperation and Consultation			Direct clinical practice			Professional development			Ethical decisions‐ making			Critical thinking		
	*F*	β^a^	p*	*F*	β^a^	p*	*F*	β^a^	p*	*F*	β^a^ *R* ^2^ 0.141	p*	*F*	β^a^ *R* ^2^ 0.184	p*	*F*	β^a^ *R* ^2^ 0.074	p*
Managerial responsibility							7.545		0.007*	6.618		0.011*				6.339		0.012*
No managerial responsibility								−0.0613 0^b^			6.945^c^ 0^b^						−0.386 0^b^	
Managerial responsibility/Academic degree							4.751		0.010*							3.326		0.038*
Managerial responsibility/Bachelor degree								5.401									2.231	
No managerial responsibility/bachelor degree								0^b^									0^b^	
Managerial responsibility/Master 60 credits								20.670^c^									4.000^c^	
No managerial responsibility/Master 60 credits								0^b^									0^b^	
Managerial responsibility/educational background										3.676		0.027*						
Managerial responsibility/2‐year direct education and no postgraduate in theatre care											−5.246							
No managerial responsibility/ 2‐year direct education and no postgraduate in theatre care											0^b^							
Managerial responsibility/RN education with 1‐year postgraduate in theatre care											−7.361^c^							
No managerial responsibility/RN education with 1‐year postgraduate in theatre care											0^b^							
Managerial responsibility/RN education with 1‐year advanced nursing in theatre care											0^b^							
No managerial responsibility/RN education with 1‐year advanced nursing in theatre care											0^b^							

Note

*Significant level at *p*‐value > 0.05, measured by ANOVA analysis.

^a^ Regression coefficient.

^b^ This parameter is set to zero because it is redundant.

^c^ Fisher's LSD test.

#### 4.1.2 Need for competence development in managerial responsibility

Operating theatre nurses with managerial responsibility and need for competence development emerged as being statistically significant related to an academic degree (Table 4). Participants with managerial responsibility and an academic degree had lower need for competence development in *Cooperation and Consultation* as did OTNs with master's degree of 60 credits in advanced nursing in theatre care (α = 0.001). In general, OTNs with master's degree of 60 credits rated need for competence development slightly lower than those with a bachelor's degree in *Professional development and Critical thinking*.

**TABLE 4:** Managerial responsibility in relation to need of competence development (*n* = 80)

The six components of the modified PROFFNurse SAS in perioperative nursing	Clinical leadership			Cooperation and consultation			Direct clinical practice			Professional development			Ethical decisions‐ making			Critical thinking		
Demographic background (*n*)	*F*	β^a^ *R* ^2^ 0.631	p*	*F*	β^a^ *R* ^2^ 0.710	p*	*F*	β^a^ *R* ^2^ 0.699	p*	*F*	β^a^ *R* ^2^ 0.141	p*	*F*	β^a^ *R* ^2^ 0.281	p*	*F*	β^a^ *R* ^2^ 0.599	p*
Academic degree				6.236		0.004*				3.504		0.039*				4.786		0.012*
Bachelor degree (20)					−75.808^c^						−63.269^c^						13.478^c^	
Master degree (60 credits) (14)					−120.641^c^						−70.782^c^						−22.017^c^	
No academic degree (53)					0^b^						0^b^						0^b^	
Educational background	5.534		0.007*				11.344		0.001*	10.499		0.001*				6.606		0.003*
2‐year direct education and no postgraduate in Theatre care (42)		25.653^c^						31.513			37.967^c^						13.427^c^	
RN education with 1‐year postgraduate in Theatre care (28)		31.170^c^						22.180			47.324^c^						13.737^c^	
RN education with 1‐year advanced nursing in Theatre care (18)		0^b^						0^b^			0^b^						0^b^	
Professional experience in perioperative nursing	10.621		0.001*	10.567		0.001*	8.502		0.001*	11.909		0.001*				13.893		0.001*
<10 years (13)		3.414			0.504			27.748^c^			14.036^c^						4.006	
10–20 years (30)		5.469			16.020^c^			40.045^c^			18.643^c^						11.422^c^	
>20 years (46)		0^b^			0^b^			0^b^			0^b^						0^b^	
Experience of managerial responsibility	29.174		0.001*	10.627		0.002*	15.129		0.001*	25.209		0.001*	5.929		0.018*	16.426		0.001*
<5 years of experience (44)		7.871			57.705^c^			−0.782			13.116			−8.731			8.651	
>5 years of experience (46)		0^b^			0^b^			0^b^			0^b^			0^b^			0^b^	
Place of employment	6.490		0.003*	4.255		0.020*	10.254		0.001*	6.486		0.003*						
University hospital		54.474^c^			66.009^§^			216.740^c^			61.699^c^							
Central/Regional hospital		−17.338^c^			−14.238			−18.813			−29.228							
District hospital		0^b^			0^b^			0^b^			0^b^							

Note

*Significant level at *p*‐value > 0.05, measured by ANOVA analysis.

^a^ Regression coefficient.

^b^ This parameter is set to zero because it is redundant.

^c^ Fisher's LSD test.

Operating theatre nurses educational background related to experience of managerial responsibility showed statistical significance related to *Clinical leadership, direct clinical practice, professional development and critical thinking*. Participants with RN education and 1‐year advanced nursing in theatre care had a lower need for competence development than other participants.

Professional nursing experience in perioperative nursing was shown to have significance in OTNs managerial responsibility, where OTNs with >20 years’ experience had a lower need for competence development compared to other participants. On the other hand, participants with 10–20 years of professional experience had an increased need for competence development in *direct clinical practice* and *critical thinking*. What was surprising was that participants <10 years of professional experience had less need for competence development compared to participants with 10–20 years of professional experience (α = 0.001) in *Cooperation and consultation*.

In experience from managerial responsibility statistical significance emerged in all components (Table 4). Participants with <5 years of experience in managerial responsibility had more need for competence development in cooperation and consultation (α = 0.044) compared with participants with >5 years. On the other hand, it emerged that those with <5 years had a lower need for competence development in *Ethical decisions making* and *direct clinical practice* compared with those with >5 years.

Another background variable of significance for OTNs managerial responsibility was place of employment when it came to need for competence development. OTNs working in the university hospital (α = 0.001) had a greater need for competence development in *Direct clinical practice*. There was also a need for competence development in *Clinical leadership* (α = 0.005)*, cooperation and consultation* (*α = 0.026)* and *professional development* (α = 0.011) compared with other participants.

### 4.2 Interactions between background variables and experience of managerial responsibility

The background factors of academic degree, educational background, professional experience in perioperative nursing, place of employment and age in interaction with experience of managerial responsibility were shown to be significant for need for competence development (Table 5). All participants with <5 years’ experience of managerial responsibility, with an academic degree, needed competence development compared with those with >5 years. Thus, in *ethical decisions‐making,* participants without an academic degree had a lower need compared with those with an academic degree. Another factor that was important for managerial responsibility and need for competence development was the education to become an OTN. In *cooperation and consultation*, all participants with <5 years’ experience of managerial responsibility, regardless of educational background, had need for competence development. OTNs with 2‐year direct education and no postgraduate education in theatre care needed more competence development in *clinical leadership and critical thinking* compared with others.

**TABLE 5:** Significant interactions between OTNs managerial responsibility and background factors (*n* = 80)

	Clinical leadership			Cooperation and consultation			Direct clinical practice			Professional development			Ethical decisions‐ making			Critical thinking		
Demographic background	*F*	β^a^ *R* ^2^ 0.631	p*	*F*	β^a^ *R* ^2^ 0.710	p*	*F*	β^a^ *R* ^2^ 0.699	p*	*F*	β^a^ *R* ^2^ 0.653	p*	*F*	β^a^ *R* ^2^ 0.281	p*	*F*	β^a^ *R* ^2^ 0.599	p*
Academic degree/Experience of managerial responsibility	10.150		0.001*	17.321		0.001*	6.136		0.005*	11.015		0.001*	4.280		0.019*	8.471		0.001*
Academic degree, Bachelor degree/< 5 years’ experience of managerial responsibility		21.295^c^			32.872^c^			58.689^c^			27.625^c^			41.819^c^			16.237^c^	
Academic degree, Bachelor >5 years’ experience of managerial responsibility		0^b^			0^b^			0^b^			0^b^			0^b^			0^b^	
Academic degree, Master 60 credits/<5 years’ experience of managerial responsibility		25.761^c^			52.665^c^			52.999^c^			34.150^c^			37.611^c^			13.047^c^	
Academic degree, Master 60 credits/>5 years’ experience of managerial responsibility		0^b^			0^b^			0^b^			0^b^			0^b^			0^b^	
No academic degree/<5 years’ experience of managerial responsibility		0^b^			0^b^			0^b^			0^b^			0^b^			0^b^	
No academic degree/>5 years experience of managerial responsibility		0^b^			0^b^			0^b^			0^b^			0^b^			0^b^	
Educational background/experience of managerial responsibility	5.719		0.006*	5.283		0.009*				5.977		0.005*				8.291		0.001*
2‐year direct education, no postgraduate in theatre care/<5 years’ experiences of managerial responsibility		−12.296			−13.281						−15.019						−8.446	
2‐year direct education, no postgraduate in theatre care/>5 years’ experience of managerial responsibility		0^b^			0^b^							0^b^					0^b^	
RN with 1‐year postgraduate in theatre care/<5 years’ experience of managerial responsibility		−24.011^c^			28.316^c^						−29.760^c^						−20.048^c^	
RN with 1‐year postgraduate in theatre care/>5 years’ experience of managerial responsibility		0^b^			0^b^						0^b^						0^b^	
RN with 1‐year advanced nursing in theatre care/<5 years’ experience of managerial responsibility		0^b^			0^b^						0^b^						0^b^	
RN with 1‐year advanced nursing in theatre care/>5 years’ experience of managerial responsibility		0^b^			0^b^						0^b^						0^b^	
Professional experience in perioperative nursing/experience of managerial responsibility	4.181		0.047*															
<10 years/<5 years		0																
>10 years/>5 years																		
10–20 years/<5 years		12.835^c^																
10–20 years/>5 years		0^b^																
>20 years/<5 years		0^b^																
>20 years/>5 years		0^b^																
Place of employment/Experience of managerial responsibility/				3.878		0.028*												
University hospital/>5 years					22.254^c^													
Regional hospital/<5 years					0^b^													
Regional hospital/>5 years					17.493^c^													
District hospital/ < 5 years					0^b^													
District hospital/>5 years					0^b^													
Age/Experience of managerial responsibility				5.559		0.023*												
Age/<5 years					−1.255^c^													
Age/>5 years					0^b^													

Note

*Significant level at *p*‐value > 0.05, measured by ANOVA analysis

^a^ Regression coefficient.

^b^ This parameter is set to zero because it is redundant.

^c^ Fisher's LSD test.

Having professional experience in perioperative nursing was shown to be of importance for managerial responsibility, where OTNs with >20 years’ experience had a lower need for competence development. However, participants with <5 years’ experience of managerial responsibility and 10–20 years of professional experience in perioperative nursing (α = 0.047) were shown to need more competence development in clinical leadership. Also, the place of employment was important in need for competence development in *cooperation and consultation*. OTNs employed at university hospitals with <5 years of experience of managerial responsibility needed more competence development compared with participants at regional/county and district hospitals. It also emerged that OTNs with managerial responsibility needed competence development in *cooperation and consultation,* but it decreased with increasing age (α = 0.023).

## 5 DISCUSSION

The study investigated and compared OTNs with and without managerial responsibility regarding clinical competence and their need for competence development. This study shows that OTNs with managerial responsibility with master's degree of 60 credits and RNs with 1 year in advanced nursing in theatre care, self‐rated themselves higher when it came to *direct clinical practice, professional development* and *critical thinking*. Aiken et al. (2014, 2017) found in their study conducted in Europe that hospitals with a higher proportion of nurses with academic degrees reported a higher degree of quality of care and a better patient safety culture. The results showed that OTNs with an academic degree and education at advanced level have a critical approach and can evaluate and handle various complex situations. This was also proven by another study (Blomberg et al., 2019). Banschbach (2016) explains that people are not always born to be leaders, and perioperative leaders must understand power issues, must have executive presence and succession planning. Within perioperative practice, it is usual for OTNs with long professional experience to be recruited to assignments with managerial responsibility. These assignments demand that OTNs must be able to strategically plan and organize allocated resources to guarantee patient safety (McCallin & Frankson, 2010). Pilat and Merriam (2019) highlighted that expectations, essential knowledge and skills, graduate education prepared, sought support and mentoring from colleagues and role mastery is not possible. That indicate that there is a lack of education and support in the transition from being clinically active to having managerial responsibility and few onboarding programmes targeted to nurse leader and it is important in their upcoming role as leader. Huston (2008) was quick to emphasize that nursing education programmes and healthcare organizations must prepare nurses to be effective leaders. Leadership is a central part of nursing and this can be achieved through incorporating leadership learning into clinical skills practice as a continuous theme as soon as RN education starts (Francis‐Shama, 2016).

This study showed that OTNs with master's degree of 60 credits and RNs with 1 year in advanced nursing in theatre care also rated themselves high in *direct clinical practice*. In this study, 70% of the participants had different managerial responsibility and almost worked clinically in perioperative practice, although 30% were first‐line managers. According to Sørensen et al. (2011), first‐line level leadership is characterized as a balance between proximity and distance to clinical activities. This contrasts to the clinical role based solely on professional experience without theoretical basis, where no development takes place in clinical activities. In the managerial role, a conflict arose between clinical and administrative work. This contrasts the hybrid role where there was an understanding of the use of knowledge based on research. This may indicate that OTNs who have managerial responsibility in this study were, according to Sørensen et al. (2011), in a hybrid role. Blomberg et al. (2019) also showed that OTNs without an academic degree wanted competence development in scientific knowledge to be able to participate in quality and improvement work. It also emerged that OTNs’ scientific competence was not utilized to develop perioperative practices. First‐line managers without an academic degree should, therefore, see as an advantage to have OTNs with scientific competence.

Need for competence development in managerial responsibility showed that OTNs with master's 60 credits had a lower need for *consultation and cooperation*. They generally rated themselves lower in *professional development* and *critical thinking* than OTNs with a bachelor's degree. Guo et al. (2021) show that nurses use a more promoting and constructive voice when they think scientifically and reason logically. Nurses possessing critical thinking can have the ability to make more advanced decisions. The same was shown when participants with <10 years of professional experience in perioperative nursing had a lower need for competence development in consultation and cooperation than OTNs with 10–20 years. Perhaps this can be explained by the fact that they have an academic degree.

On the other hand, it emerged that OTNs with <5 years of experience from managerial responsibility have an increased need for competence development in *consultation and cooperation*. There are expectations that new nursing leaders have the skills required to carry out assignments. A study of Pilat and Merriam (2019) showed that the organization needs to invest time and financial resources, as well as offer support to new nursing leaders. New nursing leaders sought the support of other leaders to understand the ethics behind what they do as leaders. A lack of clear expectations, essential knowledge and skills can lead to role insufficiency. Doyle (2018) also shows that to be able to use situational learning and critical thinking while providing leadership, mentoring and support are required. Gundrosen et al. (2016) add that there is a need for continuous support both administrative and emotional, along with proper education throughout their time as leaders. OTNs highlighted the need for interprofessional learning with colleagues working with other surgical specialties (Blomberg et al., 2019) and it can also be an option for OTNs with less experience in managerial responsibility. In this study, it emerged that OTNs with 2‐year direct education and no postgraduate education in theatre care rated themselves lower in *clinical leadership* and *critical thinking* and need support as they start to become nurse managers.

In terms of educational background, RN participants with 1‐year advanced nursing in theatre care had a lower need for competence development in *clinical leadership, direct clinical practice, professional development* and *critical thinking* than others. This can also be attributed to the fact that they have an academic degree. This is also confirmed by Guo et al. (2021) who believe that nurses with leadership usually accept constructive criticism and take appropriate actions before rigorous assessment of their abilities. Another problem is the loss of experienced OTNs with critical thinking abilities caused by some focusing on managerial responsibility. This directly affects the quality of care and patient safety provided. It also emerged that participants with managerial responsibility employed in university hospitals need competence development in *direct clinical practice* compared with others employed at regional/central hospitals. This is interesting at university hospitals, where more specialized care is performed and OTNs are faced with greater challenges. This places demands on those with a managerial responsibility.

The background factor of experience from managerial responsibility was also tested regarding interactions between other background factors. It showed that all participants with <5 years’ experience and with an academic degree needed competence development compared with those with >5 years. It emerged that regardless of educational background, there was a need for competence development in *consultation and cooperation* among OTNs with <5 years’ experience from managerial responsibility. Being able to communicate is, according to Plonien (2015), the key that opens the door to accomplishment. It is important that the leader adapts his/her way of communicating, both listening and speaking. How the leader conveys and changes communication determines their ability to motivate the team to work more effectively. It also emerged in the study that participants with longer experience in perioperative nursing had less need for competence development in *clinical leadership* than those with >5 years’ experience of managerial responsibility and 10–20 years. This suggests that, according to Pilat and Merriam (2019), OTNs with less experience in perioperative nursing need more support and education as they transition into a managerial responsibility. Although the result showed that OTNs with managerial responsibility need competence development, it decreased with increasing age.

### 5.1 Limitations

Just 30% of the 303 OTNs who participated in the study indicated that they had managerial responsibility (Holbrook et al., 2007). This can be considered low but can still give an idea of how OTNs estimate clinical competence and need of competence development in relation to managerial responsibility. The questionnaire could be answered both via email and mobile phone, which was likely to be beneficial to OTNs. How they indicated what managerial responsibility they had, turned out to be unclear in the questionnaire, and some participants may have missed this. During ongoing data collection, there were also organizational changes, usually involving OTNs with managerial responsibility. This can also be an additional reason for non‐response. The questionnaire chosen for the study had not been psychometrically tested in perioperative nursing but was pilot tested before the study started by OTNs working in different operating theatres in Sweden. The participants’ average age was high, which can be explained by the fact that most OTNs having managerial responsibility have long professional experience from perioperative nursing. It would have been interesting with younger participants and might have influenced the results of the study. A calculation from the National Board of Health and Welfare (2018) showed that there is a shortage of specialized nurses, including OTNs, and 85% of employers need to employ specialized nurses. In the future, these should be able to have managerial responsibility in perioperative care.

## 6 CONCLUSION

The study shows that OTNs with managerial responsibility and an academic degree, and RNs with 1year advanced nursing in theatre care have a critical approach and can evaluate and handle various complex situations. It also shows that OTNs with <5 years’ managerial experience have more need for competence development and especially if they were employed at a university hospital. It also emerged that they need support from more experienced OTNs with managerial responsibility, need education in leadership and consultation and cooperation from experienced colleagues at the start. They then need continuous competence development to be able to make the right decisions in different situations that apply to employees and ensure patient care. Further longitudinal studies on OTNs with managerial responsibility regarding their continuous development of competence and need of competence development are of interest.

## CONFLICT OF INTEREST

The authors declare that they do not have any conflicts of interest.

## ETHICAL CONSIDERATIONS

The study was approved by the local university committee (2015/722). Ethical standards of research were followed in accordance with Declaration of Helsinki (2013).

## Data Availability

The data that supports the findings of this study are available in the supplementary material of this article.
